# Limitations to implementing alcohol screening with an electronic clinical reminder in the Veterans Affairs health-care system: a qualitative study

**DOI:** 10.1186/1940-0640-7-S1-A94

**Published:** 2012-10-09

**Authors:** Emily Williams, Carol Achtmeyer, Rachel Thomas, Joel Grossbard, Gwen Lapham, Laura Johnson, Evette Ludman, Douglas Berger, Katharine Bradley

**Affiliations:** 1 Veterans Affairs Puget Sound and the Department of Health Services, University of Washington, Seattle, WA, USA; 2 Veterans Affairs Puget Sound and the Department of Psychiatry, University of Washington, Seattle, WA, USA; 3 Group Health Cooperative, Veterans Affairs Puget Sound, University of Washington, Seattle, WA, USA; 4 Veterans Affairs Puget Sound and the Department of Medicine, University of Washington, Seattle, WA, USA

## 

Implementation of alcohol screening and brief intervention (SBI) is a prevention priority. The Veterans Affairs (VA) Healthcare System uses a clinical reminder (CR) in the electronic medical record to prompt and document results of screening and trigger a subsequent CR for BI when screening is positive. Although screening rates are over 90%, marked variability in screening quality has been documented. Four researchers observed clinician interactions with CRs during alcohol screening at nine primary care clinics in the northwest US to identify barriers and facilitators to using CRs to implement quality screening. Observers took handwritten notes, which were transcribed and analyzed qualitatively using an a priori coding template adapted during analyses. We observed 58 support staff (25 registered nurses, 26 licensed practical nurses, and seven health technicians) caring for 166 patients. Alcohol screening prompted by the CR was often uncomfortable and of low quality. Clinicians often offered disclaimers prior to screening or made adjustments to how questions were presented, with some citing the sensitive nature of the questions. Verbal screening typically did not include asking questions verbatim. There was substantial variability in methods of conducting screening across clinics, with some using the CR to facilitate in-person screening by interview and others entering patient responses into the CR after completion of a paper-based screen. Although the CR was designed to trigger a subsequent CR for BI when positive, some clinics used paper encounter forms for this. Findings suggest that VA CRs have important limitations as a method of facilitating effective, high-quality alcohol screening. Barriers observed reflect a combination of limitations of CR technology (and the alcohol screening CR specifically), ways the CR was implemented, clinical workflow, complexity of patient needs, and alcohol-related stigma. Future research should address these barriers to effectively implement recommended care.

